# Clinical characteristics, HPV involvement, and demographic risk factors in women with cervical intraepithelial neoplasia complicated by vaginal intraepithelial neoplasia

**DOI:** 10.1186/s12905-024-03030-1

**Published:** 2024-04-04

**Authors:** Mindan Xu, Yan Wang

**Affiliations:** Department of Obstetrics and Gynecology, First affiliated Hospital of Soochow University, 188 Shizi Street, Suzhou, Jiangsu 215000 People’s Republic of China

**Keywords:** Cervical intraepithelial neoplasia, Vaginal intraepithelial neoplasia, Human papillomavirus, ThinPrep cytologic test

## Abstract

**Purpose:**

This study aimed to explore the clinical characteristics and risk factors associated with cervical intraepithelial neoplasia (CIN) when coexisting with vaginal intraepithelial neoplasia (VAIN).

**Methods:**

We analyzed the clinical data of 212 patients diagnosed with CIN, including 50 patients with concurrent VAIN. The groups were compared to identify distinct clinical features and independent risk factors for the co-occurrence of CIN and VAIN, using logistic regression analysis.

**Results:**

Patients with both CIN and VAIN had a median age of 57, significantly older than the 41-year median age of patients with CIN only (*P* < 0.05). A higher prevalence of HPV infection (98.0%) was observed in the CIN and VAIN group, with a notable rate of multiple HPV infections (67.3%) compared to the CIN-only group (*P* < 0.05). Educational levels were significantly lower in the combined CIN and VAIN group (*P* < 0.05). HPV16, 33, and 52 were identified as significant types for single and multiple infections. Multivariate analysis confirmed age as an independent risk factor for CIN with VAIN (*P* < 0.05). VAIN3 patients were more likely to exhibit HSIL and ASC-H, whereas VAIN1 cases tended to correspond with ASCUS and LSIL diagnoses.

**Conclusion:**

The co-occurrence of CIN and VAIN is significantly influenced by patient age and educational level. The findings advocate for more diligent vaginal examination during colposcopy in older patients, particularly those with multiple HPV infections and cytological abnormalities, to enhance the early detection of vaginal lesions and prevent missed diagnoses and treatments. Additionally, the high prevalence of HPV infection, especially with certain types, underscores the importance of HPV monitoring in this patient population.

## Introduction

Vaginal intraepithelial neoplasia (VAIN) represents a precancerous condition of the vagina, characterized by epithelial dysplasia and carcinoma in situ without invasion beyond the basal membrane [1]. This condition, largely attributed to human papillomavirus (HPV) infection, is classified into low-grade and high-grade intraepithelial lesions based on the differentiation potential of squamous cells and associated clinical risks. Low-grade lesions, featuring mature and differentiable squamous cells, pose a lesser risk of recurrence or progression to invasive cancer. In contrast, high-grade lesions are marked by the proliferation of immature squamous epithelial cells post-HPV infection, which, if untreated, are more likely to recur or develop into invasive cancer.

VAIN predominantly affects women aged 35 to 55[2]. Although it is a relatively rare condition with an incidence of only 0.2 to 0.3 per 100,000, it is important to note that the number of VAIN cases represents approximately 0.6–1% of the total number of cervical intraepithelial neoplasia (CIN) cases (3, 4). This comparison highlights the lesser prevalence of VAIN compared to CIN, yet underscores its significance as part of the spectrum of female lower reproductive tract intraepithelial neoplasias. The low incidence of VAIN could be attributed to its often asymptomatic nature and reliance on singular diagnostic methods. A colposcopic examination should be performed with the application of 5% acetic acid and then a colposcopically guided biopsy may be performed [5]. Recent studies, however, indicate a rising incidence of VAIN, particularly among patients with cervical lesions. This trend may be linked to high-risk HPV infections or persistent infections, alterations in the vaginal microenvironment, and the severity of concurrent cervical lesions. Moreover, the treatment approach for cervical lesions appears to influence VAIN incidence, with a noted increase in VAIN cases in the vaginal stump post-hysterectomy [6].

Despite these insights, VAIN often goes undetected due to its lack of specific symptoms, the oversight of the vaginal site in colposcopic diagnoses, limited disease awareness, and small-scale studies. This situation underscores the need for heightened vigilance and comprehensive examination strategies to improve VAIN detection and management. This study aimed to explore the clinical characteristics and risk factors associated with cervical intraepithelial neoplasia (CIN) when coexisting with vaginal intraepithelial neoplasia (VAIN).

## Materials and methods

### Study population

This study involved 212 patients diagnosed with cervical intraepithelial neoplasia (CIN) from May 2019 to May 2023. Among these, 50 patients were diagnosed with CIN complicated by vaginal intraepithelial neoplasia (VAIN), while the remaining 162 had CIN only. Inclusion Criteria: Patients were included if they underwent a cervical or vaginal wall biopsy via colposcopy at our hospital, with subsequent histopathological confirmation of CIN or VAIN. Exclusion Criteria: Patients were excluded if they had a current or past history of any malignant tumor, uterine prolapse, endometrial atypical hyperplasia, or other significant gynecological diseases. Clinical data were collected for all enrolled patients. This included age, clinical symptoms, menopausal status, smoking history, marital history, results from the ThinPrep cytologic test (TCT), and the grading of CIN and VAIN.

### TCT

Procedure: For the TCT, exfoliated cells were collected from the cervical canal and cervicovaginal area. Following sample preparation, Pap staining was applied, and a pathologist performed the diagnostic evaluation. Classification: The results were categorized according to the 2014 Bethesda classification of cervical cytology (TBS) reporting system, also known as the Bethesda 3-tier system. The categories included: (1) Negative for intraepithelial lesion or malignancy (NILM) (2) Atypical squamous cell of undetermined significance (ASCUS); (3) Atypical squamous cells-cannot exclude HSIL (ASC-H); (4) Low-grade squamous intraepithelial lesion (LSIL); (5) High-grade squamous intraepithelial lesion (HSIL).

### HPV detection

HPV detection was performed using microfluidic automatic nucleic acid detection technology (qiagen, Germany). Specimen extraction was facilitated by an HPV detection kit, and a nucleic acid chip detector was employed for identifying HPV types. The detection covered a range of HPV types, categorized into high-risk and low-risk groups: High-risk types: 16, 18, 31, 33, 35, 39, 45, 51, 52, 53, 56, 58, 59, 66, 68, 73, 82, and 83.Low-risk types: 6, 11, 42, 43, 44, and 81.

### Histopathological examination

Procedure: The diagnosis of cervical and vaginal lesions was established based on the histopathological findings from cervical and vaginal wall biopsies obtained during colposcopy. Classification of CIN (Cervical Intraepithelial Neoplasia): According to the 2014 WHO classification of female genital organ tumors, CIN is categorized into: LSIL: Includes CIN1 and immunochemically P16-negative CIN2. HSIL: Comprises CIN3 and P16-positive CIN2. Classification of VAIN (Vaginal Intraepithelial Neoplasia): Histologically, VAIN is divided into: LSIL (VAIN1) and HSIL (VAIN2-3).

### Statistical analysis

Software Used: The data were analyzed using SPSS version 19.0 statistical software. Analysis Methods: Quantitative data that conformed to a normal distribution were expressed as mean ± standard deviation (x ± s) and analyzed using the t-test and logistic regression analysis. For comparisons among groups, the chi-square test (χ2 test) was utilized. Significance Threshold: A p-value of less than 0.05 (*P* < 0.05) was considered to indicate statistical significance.

## Results

### General information

Table 1 presents a comparison of the general information and clinical data between the CIN group and the CIN combined with VAIN group. This comparison includes variables such as age, smoking status, alcohol consumption, HPV status, age at first sexual intercourse, number of sexual partners, number of pregnancies, and educational level.

**Table 1:** Comparison of baseline clinical data between CIN group and CIN combined with VAIN group

Parameters	CIN group (*n* = 162)	CIN combined with VAIN group (*n* = 50)	*P*
Age	41.30 ± 5.59	57.40 ± 9.41	< 0.001
Smoking status	6(3.7%)	2(4.0%)	0.987
Alcohol status	8(4.9%)	3(6.0%)	0.844
HPV status			0.001
Negative	5(3.1%)	1(2.0%)	
single infection	100(61.7%)	16(32.0%)	
Multiple infection	57(35.2%)	33(66.0%)	
Age of first sexual intercourse	21.62 ± 1.52	21.46 ± 1.59	0.527
Number of sexual partners			0.151
1	156(96.3%)	48(96.0%)	0.924
≥ 2	6(3.7%)	2(4.0%)	
Number of pregnancies			0.648
1	140(86.4%)	42(84.0%)	
≥ 2	22(13.6%)	8(16.0%)	

CIN, maintain hemodialysis; VAIN, Body Mass Index

### Significant findings

Notably, significant differences were observed between the two groups in terms of age, HPV status, and educational level (*P* < 0.05).

### HPV types

HPV16 infection was the most prevalent in both groups. In the CIN combined with VAIN group, the most common infections, in order of frequency, were HPV16 (22 cases), followed by HPV18, HPV52, HPV33, and HPV56. In the CIN group, HPV16 led with 71 cases, followed by HPV18, HPV56, HPV33, and HPV52. Notably, the rates of HPV16, HPV33, and HPV52 infection were significantly lower in the CIN group compared to the CIN with VAIN group (*P* < 0.05, as detailed in Table 2).

**Table 2:** Common HPV types for CIN group and CIN combined with VAIN group

Parameters	CIN group (*n* = 162)	CIN combined with VAIN group (*n* = 50)	P
HPV16			0.026
Single infection	46(64.8%)	8(36.7%)	
Multiple infection	25(35.2%)	14(63.3%)	
HPV18			0.269
Single infection	16(57.1%)	3(33.3%)	
Multiple infection	12(42.9%)	6(66.7%)	
HPV33			0.031
Single infection	13(86.7%)	2(32.0%)	
Multiple infection	2(13.3%)	4(66.0%)	
HPV52			0.041
Single infection	11(78.6%)	2(28.6%)	
Multiple infection	3(21.4%)	5(71.4%)	
HPV56			0.615
Single infection	8(33.3%)	1(25.0%)	
Multiple infection	16(66.7%)	3(75.0%)	

CIN, cervical intraepithelial neoplasia; VAIN, vaginal intraepithelial neoplasia

### TCT results

Among patients with VAIN3, all TCT results were abnormal. Specifically, 81.8% of these patients exhibited either HSIL or ASC-H, a proportion that was significantly different from patients with VAIN1. Further details and comprehensive data can be found in Table 3.

**Table 3:** Analysis of TCT results in VAIN patients

TCT	VAIN1(*N* = 30)	VAIN2(*N* = 9)	VAIN3(*N* = 11)	P
NILM	8(26.7%)	1(11.1%)	0(0%)	< 0.001
ASCUS	14(46.7%)	2(22.2%)	1(9.1%)	
LSIL	7(23.3%)	3(33.3%)	1(9.1%)	
HSIL	1(3.3%)	3(33.3%)	5(45.5%)	
ASC-H	0(0%)	0(0%)	4(36.4%)	

NILM, negative for intraepithelial lesion or malignancy; ASCUS, Atypical squamous cell of undetermined significance; LSIL, Low-grade squamous intraepithelial lesion; HSIL, High-grade squamous intraepithelial lesion; ASC-H, Atypical squamous cells-cannot exclude HSIL

## Discussion

With the widespread implementation of cervical cancer screening programs, a significant number of precancerous cervical lesions are being detected and effectively treated, leading to favorable prognoses. VAIN, characterized histologically by dysplasia of the vaginal epithelium without stromal invasion, is one such lesion that can be reliably diagnosed [3]. Although VAIN and CIN share similar risk factors, the incidence of CIN is notably higher than that of VAIN [4]. However, in recent years, advancements in cytology and HPV detection, combined with increased vigilance by colposcopists towards vaginal lesions, have led to a rise in the detection rates of VAIN [3].

VAIN and CIN represent precancerous lesions of the vaginal and cervical epithelia, respectively. Given that these tissues are homologous, some studies suggest that VAIN may be an extension of CIN. Research on patients with CIN and cervical cancer indicates a significant overlap, with up to 14.2% (99 out of 699 patients) presenting with concurrent VAIN [7]. Furthermore, data reveal that 72.7% of these patients have VAIN, and those with CIN or cervical cancer are 82 times more likely to develop VAIN compared to those without these conditions [8]. The severity of VAIN is closely related to the level of CIN or cervical cancer. Generally, a higher grade of CIN correlates with a higher incidence and severity of VAIN. Interestingly, some studies have found no significant difference in the age or timing of VAIN development post-surgery in patients with high-grade CIN or cervical cancer, suggesting that the occurrence of VAIN post-surgery is not age-dependent [9]. Moreover, there appears to be a correlation between the grades of VAIN and CIN. Specifically, when the incidence of CIN I and CIN III is higher than that of CIN II, a similar pattern is observed in VAIN, with the number of VAIN I and VAIN III cases surpassing those of VAIN II. This suggests a parallelism in the distribution of lesion severity between the cervical and vaginal epithelia.

Over 200 HPV types have been identified, with more than 40 known to infect the cervix. HPV types vary in terms of the tissue they infect and their associated risk. For skin infections, HPV types can be categorized into low-risk (e.g., 1, 2, 3, 4, 7, 10, 12, 15) and high-risk types (e.g., 5, 8, 14, 17, 20, 36, 38) [3]. The International Agency for Research on Cancer (IARC) of WHO distinguishes 3 groups of HPV. HR genotypes include HPV classified into group 1 (HPV 16, 18, 31, 33, 35, 39, 45, 51, 52, 56, 58, 59) and group 2 A (HPV 68) [10]. According to the IARC, 13 HPV genotypes are currently classified into the HR HPV group; HPV genotypes 16, 18, 31, 33 and 45 are considered the most important for the development of human malignancies [10]. Low-grade VAIN is commonly associated with both high-risk HPV subtypes (16, 18, 31, 33, 35, 45, 51, 52, 56) and low-risk types (6, 11, 42, 43, 44). In cases of high-risk HPV infection, there is often co-infection with subtypes 16 and 18, accounting for more than 50% of these cases [3, 5]. Furthermore, some studies indicate that vaginal lesions post-hysterectomy for high-grade CIN are predominantly high-grade or invasive, with HPV binding sites in these vaginal or vulvar tissues being consistent with those found in previous cervical lesions (11, 12).

Our study’s findings emphasize the significant association between the co-occurrence of CIN and VAIN with patient age and educational level. This underscores the critical need for thorough vaginal examinations during colposcopy, especially in older patients or those with multiple HPV infections and cytological abnormalities, to facilitate early detection and treatment of vaginal lesions [13–16].

The current trends in bioinformatics and meta-analysis offer promising tools for future research in CIN and VAIN [17–30]. There is a notable gap in the application of these advanced analytical methods in understanding the genetic and molecular aspects of these conditions. Future research should focus on exploring gene expression levels, transcriptomics, proteomics, and genetic polymorphisms associated with vaginal intraepithelial neoplasia. Such investigations could provide valuable insights for developing more precise diagnostic and therapeutic strategies. It is worth mentioning that the adoption of HPV vaccination in patients having treatment for HPV-related disease. Even in the absence of the uterine cervix, HPV vaccination would protect against develop lower genital tract dysplasia [31]. Furthermore, many factors have been discussed in CIN in the past years, which have been reported by multiple literatures. However, the factors are multifarious and complicated. The latest evidence shows that surgical treatment of the CIN was associated with an increased risk of preterm delivery, lower birth weight and preterm premature rupture of membrane before 37 pregnancy weeks compared to untreated women, especially in a Cold-Knife Conization (CKC) and Large Loop Excision of Transformation Zone (LLETZ) procedure [32]. Moreover, the increase of preterm delivery was associated with cone size, cervical length, repeated treatment and a short conization-to-pregnancy interval [33]. We should acknowledge the shortcoming of the current study. The sample size is relatively small. In future studies, we will further increase the sample size to make the results more convincing. Additionally, risk factors that influence the recurrence of high-grade cervical lesions are various [34]. It is not possible to consider all risk factors in our paper, and we will consider more in future studies. Finally, in the future study, we should investigate the long-term risk factors for the recurrence of HVP-related lesions. The latest research evidence suggests that HPV persistence is one of the most important factors predicting the risk of CIN2 + recurrence and the risk of CIN2 + recurrence increased with the increase of HPV persistence for up to 1 year [35].

## Conclusion

The co-occurrence of CIN and VAIN is significantly influenced by patient age and educational level. The findings advocate for more diligent vaginal examination during colposcopy in older patients, particularly those with multiple HPV infections and cytological abnormalities, to enhance the early detection of vaginal lesions and prevent missed diagnoses and treatments. Additionally, the high prevalence of HPV infection, especially with certain types, underscores the importance of HPV monitoring in this patient population.

## Data Availability

All data generated or analyzed during this study are included in this published article.
